# Long-term follow-up after weight management in obese cats*

**DOI:** 10.1017/jns.2014.36

**Published:** 2014-09-25

**Authors:** Gabrielle Deagle, Shelley L. Holden, Vincent Biourge, Penelope J. Morris, Alexander J. German

**Affiliations:** 1 Department of Obesity and Endocrinology, Department of Ageing and Chronic Disease, University of Liverpool, Liverpool, UK; 2 Royal Canin Research Center, Aimargues, France; 3 The WALTHAM Centre for Pet Nutrition, Freeby Lane, Waltham-on-the-Wolds, Melton MowbrayLE14 4RT, UK

**Keywords:** Weight loss, Overweight, Feline nutrition, Weight regain

## Abstract

Feline obesity is a prevalent medical disease and the main therapeutic strategy is dietary energy restriction. However, at present there are no data regarding long-term outcome in this species. The purpose of the present study was to investigate if, as in other species, some cats regain weight following successful weight loss, and to identify any influencing factors in a cohort of client-owned cats with naturally occurring obesity. Twenty-six cats were included, all of which had successfully completed a weight management programme. After weight loss, cats were periodically monitored. The median duration of follow-up was 954 d (72–2162 d). Ten cats (39 %) maintained their completion weight (±5 %), four (15 %) lost >5 % additional weight and 12 (46 %) gained >5 % weight. Seven of the rebounding cats (58 %) regained over 50 % of their original weight lost. Older cats were less likely to regain weight than younger cats (*P* = 0·024); with an approximately linear negative association between the cat's age and the amount of weight regained (Kendall's τ = −0·340, *P* = 0·016). Furthermore, cats whose energy intake during weight loss was greater were also more likely to regain weight (*P* = 0·023). When the characteristics of weight regain in cats were compared with those from a similar cohort of dogs, cats that rebounded were more likely to regain >50 % of the weight they had lost. These results suggest that weight regain, after successful weight loss, is common in obese cats, and that young cats (<7 years of age) are most at risk.

Feline obesity is a common medical disorder and predisposes to other diseases including diabetes mellitus^(^^1^^)^. Management of an overweight cat involves shifting the positive energy balance through three key components: exercise, dietary modification and owner education. Although previous studies, which use these components, have demonstrated the success of weight management strategies^(^^1^^,^^2^^)^, data are more limited on long-term outcome.

Considerable effort is required to achieve successful weight loss in cats, and to reach target weight but, arguably, maintaining that target weight is just as important. Long-term success with dietary management is poor in human subjects, with regain being common after initial weight loss. Indeed, in one meta-analysis, 29–64 % of participants regained all of the weight they originally lost and some gained additional weight^(^^3^^)^. Some studies have suggested a decreased post-weight-loss resting metabolic rate, but evidence is contradictory: a review of 12 studies determined the relative resting metabolic rate of the formerly obese group to be 5·1 % less than control^(^^4^^)^, whil another study identified only a transient hypothyroid–hypometabolic state^(^^5^^)^. In dogs, studies have identified a decreased post-weight-loss maintenance energy requirement, which had been suggested to explain the tendency for weight rebound in this species^(^^6^^–^^7^^)^. Weight regain is also seen in obese pet dogs that successfully lose weight, with one recent clinical study suggesting that almost half of dogs that reach target subsequently regain weight^(^^8^^)^. However, although weight regain has been demonstrated in experimental weight loss studies in cats^(^^9^^)^, to the authors' knowledge, no published data exist regarding the issue of regain after weight loss in obese pet cats. Therefore, the purpose of the present study was to investigate if obese, client-owned, cats maintain their weight following a successful weight loss programme, and to identify factors associated with the tendency to regain weight.

## Methods and materials

### Animals

Obese cats that had successfully completed a weight management programme at the Royal Canin Weight Management Clinic, University of Liverpool, were eligible for inclusion. All cases were recruited, investigated, and managed by the same veterinarian (A. J. G.) and veterinary nurse (S. L. H.). Cats were eligible if their weight loss had commenced between December 2004 and July 2012, and had completed by February 2013. Additional eligibility criteria were that the cats needed to have remained systemically well during their weight loss and follow-up. This was determined by the absence of clinical signs suggestive of another systemic disease, and the absence of significant abnormalities on clinicopathological analyses (routine haematology, serum biochemistry and urinalysis), throughout the period of weight loss and during follow-up. In addition, owners had to be contactable at the time of follow up. The study protocol adhered to the University of Liverpool Animal Ethics Guidelines and was approved by both the University of Liverpool Research Ethics Committee and the WALTHAM ethical review committee. The owners of all participating animals gave informed written consent.

### Weight loss phase

Complete details of the weight loss regime have been previously described^(^^2^^)^. Briefly, calibrated electronic weigh scales were used for all weight measurements, while fan-beam dual-energy X-ray absorptiometry was used to assess body composition before and after weight loss^(^^2^^)^. A tailored weight loss programme was devised for each cat, based upon dietary management (starting allocation 146–167 kJ/kg (35–40 kcal/kg) target weight/d), and lifestyle alterations. Depending upon individual preference, a high-protein high-fibre dried diet (Satiety Support; Royal Canin, Aimargues, France), a high protein dried diet (Obesity Management DP 42; Royal Canin), and/or a high protein diet (Obesity Management S/O, Royal Canin) was used. Body weight was rechecked every 14–28 d, and the weight programme was adjusted, as required to maintain steady weight loss. All adjustments to the programme were made by the same veterinary nurse (S. L. H.). On the request of the owner, one cat did not have a repeat dual-energy X-ray absorptiometry performed.

### Weight maintenance phase

After cats had reached their target weight, weight checks continued, and all monitoring (including follow-up advice) was performed by the same veterinary nurse (S. L. H.). For maintenance, owners could choose to continue using the diet food, or instead could switch to commercially available feline maintenance food of their choice, both fed to maintain bodyweight. Whichever option they chose, follow-up was similar: for cats on the diet food, at each check, food intake was increased by 5–10 % each visit, until weight loss stabilised; for cats switched to a different diet, the weight loss food was gradually substituted over 3–7 d for an equivalent amount (on an energy basis) weight loss food. For cats on either food, once stable weight was achieved, monitoring continued less frequently, and the frequency of rechecks ranged from monthly to biannually. However, additional support was offered as needed for the case, either by telephone, email or in person.

### Long-term follow-up

Two of the authors (G. D. and S. L. H.) gathered all of the follow-up information. In the early 2013, a review of all cats, that had successfully completed their weight loss programme and reached target weight, was conducted. First, the referring veterinary practice was contacted to determine whether or not cats were still registered and were still alive. If cats were still alive an attempt was then made to contact the owner to organise a reweighing. If reweighing was not possible, data from last follow-up were used. Two cats had recently developed unrelated illnesses and, as a consequence, their last healthy weight on record was used. Owners were also contacted to discuss current feeding practices. Attempts to contact owners extended over two months, and at least three attempts were made to contact each client. All except one provided current feedback. The variations of time between completion of the weight loss programme and follow-up were largely dependent on the original case enrolment date.

For the owners that were successfully contacted, information was gathered on current feeding practices, either by phone or in person. Rather than using a standardised questionnaire, open questions were asked in order to gather as much detail as possible, and points of confusion were clarified as required. An attempt was made to gather the details regarding the food fed as main meal and how much, the method of measuring food portions (e.g. use of electronic scales, measuring cups, or simply estimating portions by eye), feeding of additional food (e.g. table scraps and treats), and whether a diary record was being used. Based upon the data gathered, cats were classified as ‘regulated’ (whereby the maintenance food remained the same, food portions were measured accurately (i.e. with electronic scales), and diary records were maintained) or ‘unregulated’ (whereby different foods were used, portion size was measured in another way (commonly by estimation), and diary records were not maintained).

### Data handling and statistical analysis

All data are expressed as median (range) except where indicated. Weight regain was quantified by the percentage change from completion weight. Cats were assigned to one of three categories: ‘maintained target weight’ (*M*), if follow-up weight remained within 5 % of completion weight; ‘lost further weight’ (*L*) or ‘regained weight’ (*R*) when follow-up weight was >5 % below or >5 % above completion weight, respectively^(^^8^^)^.

Computer software (Stats Direct version 2.6.8; Stats Direct Ltd.) was used for all tests with the level of significance set at *P* < 0·05 for two-sided analyses. The Shapiro–Wilk test was used to determine whether data were normally distributed, and parametric or non-parametric tests were then used as appropriate. Simple and multiple logistical regression analysis to determine factors associated with weight regain. The outcome variable was the maintenance status of cats being either ‘regained weight/rebounded’ (greater than 5 % above target weight) or ‘not regained weight’ (equal to or less than 5 % above target weight). Factors tested included animal characteristics (e.g. age at enrolment and at follow-up, sex, target weight), weight loss characteristics (e.g. mean energy intake during weight loss, diet used for weight loss, starting body fat percentage, duration of weight loss, mean rate of weight loss, percentage weight loss, and percentage change in lean mass), environment factors (living with other cats, living with dogs, and indoor-outdoor status e.g. indoor exclusively vs. indoors with access to outdoors), and weight maintenance factors (e.g. duration of follow-up and diet control during maintenance). For the latter, based upon available records, owners were determined either to have closely regulated their cat's intake (i.e. by accurately measuring food portions, not giving table scraps or treats, and maintaining records) or had not. Given that the majority of cats were domestic shorthair (e.g. twenty-five domestic shorthair, one Selkirk Rex), and all cats were neutered, neither breed nor neuter status was assessed.

Initially, simple logistic regression was performed, whereby the effect of each factor on the outcome of interest (follow-up weight >5 % above target bodyweight) was tested individually. A multiple regression model was then constructed, which initially included any variables identified as *P* < 0·3 in simple logistic regression. The model was subsequently refined by backwards-stepwise elimination of the least significant variable at each round. Variables were retained in the final model if they were significant (*P* < 0·05) in their own right. The only exception to this approach was for factors associated with age (namely age at enrolment and age at follow-up), since these were positively correlated with one another (Kendall's τ = 0·69, *P* < 0·001), leading to a concern regarding the possible effect of confounding on the multiple regression model. As a result both factors were tested independently in the initial multiple regression model, and in combination. Given that age at enrolment had the stronger effect, and most of the effect of age at follow-up could be explained by age at enrolment, the latter was eliminated early on during the refinement process. Finally, to investigate further the reason for age differences, the Mann–Whitney test was used (to assess differences between cats <7 years of age and cats ≥7 years of age), while Fisher's exact test and Student's *t* test were used to compare regain data in cats with those of dogs using published results from a canine post-weight loss follow-up study^(^^8^^)^.

## Results

### Animals and the weight loss phase

Twenty-six cats participated in the present study (Table 1). Prior to weight loss, none of the cats had significant abnormalities on routine haematological analysis, serum biochemical analysis, and urinalysis. The median percentage weight loss was 19·8 (7·3–37·4 %) of starting body weight at a median rate of 0·6 % starting body weight per week (0·2–1·3 %) over a period of 204 d (91–796 d).

**Table 1.** Summary of cat characteristics and outcomes of weight loss and follow-up (*n* 26)

Criterion	Group summary
Breed	DSH (24), BSH (1), Selkirk Rex (1)
Sex	Neutered male (18), Neutered female (8)
Age at enrolment (months)	84 (16–156)
Age at follow-up (months)	132 (59–214)
Starting bodyweight (kg)	6·88 (5·45–10·30)
Starting body fat (%)	32 (17–45)
Diet used for weight loss*	1 (9), 2 (5), 1&3 (9), 2&3 (3)
Mean energy restriction for weight loss (kcal)	31·08 (20·78–42·16)
Duration of weight loss (days)	204 (91–796)
Bodyweight at end of weight loss (target) (kg)	5·38 (3·95–7·35)
Mean rate of weight loss (%)^†^	0·6 (0·2–1·3)
Weight loss (%)	19·8 (7·3–37·4)
Change in lean mass (%)^‡^	−6·4 (−21·5–17·5)
Other cats in household	16 yes, 10 no
Dogs in household	9 yes, 17 no
Indoor–outdoor status	3 indoor only, 23 indoor with outside access
Dietary control during maintenance^§^	Regulated (17), Unregulated (8), Unknown (1)
Bodyweight at follow-up (kg)	5·60 (3·50–10·50)
Duration of follow-up (day)^‖^	954 (72–2162)
Change from completion (target) weight (%)	2·0 (−17·5–62·8)
Regained weight (kg)	0·1 (−0·9–4·1)
Original weight loss regained (%)	7·7 (−51·4–427·3)
Status at follow-up	Maintained weight (10, 39 %), lost further weight (4, 15 %), regained weight (12, 46 %)

All data are expressed as median (range).

*Diets used for weight loss were (1) a high-protein and -fibre dried diet (Satiety Support; Royal Canin, Aimargues, France), (2) a high-protein dried diet (Obesity Management DP 42; Royal Canin) (3) a high-protein diet (Obesity Management S/O, Royal Canin). When two diets are indicated (e.g. 1&3) a combination of the two were used.

^†^Mean rate of weight loss expressed as percentage of starting bodyweight per week.

^‡^Percentage change in lean mass between the start and end of the weight loss period, where positive and negative values represent gains and losses in lean mass, respectively.

^§^For ‘dietary control during maintenance’ cats were classified as regulated (whereby the maintenance food remained the same, food portions were measured accurately (i.e. with electronic scales), and diary records were maintained) or unregulated (whereby different foods were used, portion size was measured in another way (commonly by estimation), and diary records were not maintained). It was not possible to contact one owner to acquire dietary information and, as a result the cat was classified as unknown for this variable.

^‖^Interval between the end of the weight loss phase and the review.

### Weight maintenance phase

The median duration of follow-up was of 954 d (72–2162 d). Ten cats (39 %) maintained their completion (target) weight, four (15 %) lost more weight, and the remaining 12 (46 %) regained weight. The median change from completion weight was 2·0 % (−17·5– 62·8 %), which represents 7·7 % (−51·4–427·3 %) of the original weight lost. Seven cats had regained over 50 % of their original weight lost.

### Factors associated with regain

Table 2 summarises the results from the simple and multiple regression analyses of variables associated with weight regain. On simple regression analysis, variables identified as *P* < 0·3 were age of cat at both enrolment and follow-up (*P* = 0·082 and 0·128, respectively), mean energy intake during weight loss (*P* = 0·101), having other cats in the household (*P* = 0·169) and dietary control during maintenance (*P* = 0·076). However, on multiple regression analysis, only age at enrolment and mean energy intake during weight loss were significant: older cats were less likely to regain weight than younger cats (OR 0·95; 95 % CI 0·92, 0·99; *P* = 0·024), while cats that lost weight with a greater mean energy intake were more likely to regain weight (OR 1·44; 95 % CI 1·05, 1·96; *P* = 0·023). To determine whether older cats were less likely to rebound was due to them having been followed for a shorter period, follow-up duration was compared between young (<7 years of age) and older cats (≥7 years of age): no difference was noted between groups (*P* = 0·751).

**Table 2.** Results of simple and multiple logistic regression analysis determining factors involved with tendency to rebound (*n* 26)

Logistic regression	OR	95 % CI	*P*
Initial model			
Age at enrolment (months)	0·98	0·95, 1·00	0·082
Age at follow-up (months)	0·98	0·96, 1·00	0·128
Sex	0·80	0·15, 4·24	0·793
Starting bodyweight (kg)	1·09	0·58, 2·03	0·789
Bodyweight at completion of weight loss (target) (kg)	0·91	0·32, 2·59	0·854
Mean energy restriction for weight loss (kcal)	1·17	0·97, 1·42	0·101
Diet used for weight loss – wet/dry	1·00	0·21, 4·67	>0·999
Diet used for weight loss – fibre	0·60	0·11, 3·3	0·557
Starting body fat (%)	0·94	0·84, 1·06	0·315
Duration of weight loss (days)	1·00	1·00, 1·01	0·509
Mean rate of weight loss (%)	0·19	0·01, 3·29	0·256
Weight loss (%)	1·01	0·92, 1·12	0·81
Change in lean mass (%)	0·95	0·87, 1·04	0·285
Other cats in household	0·30	0·05–1·67	0·169
Dogs in household	1·07	0·20, 5·77	0·940
Indoor–outdoor status	0·54	0·43, 6·89	0·640
Dietary control during maintenance	5·50	0·84, 36·20	0·076
Duration of follow-up (days)	1·00	1·00, 1·00	0·994
Final model			
Age at enrolment (months)	0·95	0·92, 0·99	0·0236
Mean energy restriction for weight loss (kcal)	1·44	1·05, 1·96	0·0233

For explanation of the factors, please see the legend for Table 1.

### A comparison of weight rebound in cats and dogs

Fisher's exact test demonstrated no divergence between tendency to rebound in cats *v*. dogs (*P* > 0·9999) indicating both species had a similar tendency to regain weight following weight loss. Furthermore, the weight change from target weight (*P* = 0·821) and the percentage of original weight loss regained (*P* = 0·517) were not different between dogs and cats. However, when the weight regain was expressed relative to the amount originally lost, cats were more likely to regain >50 % of their original weight loss than dogs (*P* = 0·0497).

## Discussion

Although weight regain after initial loss is well documented in obese human subjects^(^^3^^)^ and dogs^(^^7^^,^^8^^)^, the present study is the first to provide an insight into long-term outcomes of formerly obese pet cats following successful weight loss. As with pet dogs^(^^8^^)^, regain occurred in almost half of the cats originally reaching their target weight. Regarding amount of weight regain, cats are intermediate between dogs, where approximately a quarter regain 50 % or more weight^(^^7^^)^, and human subjects, where 29–64 % of the participants regain all or more of the weight they originally lost^(^^3^^)^. The reason for the difference among species is not known. In human subjects, the length of the maintenance phase is associated with rebound tendency^(^^3^^)^, but this is not a significant factor, either in dogs^(^^8^^)^, or in the cats of the present study. Further work is required to exploring the reasons for such comparative differences in long-term weight management success.

In the present study, the key factors associated with weight regain were age at enrolment and mean energy intake during weight loss. Older cats were less likely to rebound; given that the duration of follow-up was not associated with likelihood of regain, other factors must be responsible. In other studies, middle-aged cats are at greatest risk of developing obesity, but the risk strongly decreases in cats over 10 years old^(^^10^^)^. The similarity in age effect between risk of obesity and risk of regain after weight loss, suggest that the similar factors might be responsible. However, those factors are not known. In one study, cats older than 12 years had a compromised ability to digest fat and protein, which along with a decreased appetite, could contribute to this declining risk of obesity^(^^11^^)^.

A previous work in both dogs^(^^7^^)^ and cats^(^^9^^)^, has demonstrated that more marked energetic restriction during weight loss increases the risk of subsequent regain, most likely as a result of increased metabolic efficiency after weight loss. In contrast, the median energy intake (per kg of ideal weight) during the weight loss period was greater in the cats that regained weight than in those that did not. This suggests that the degree of energy restriction was actually less marked in the cats regaining weight than in those not regaining. The reason for this is not known and, in fact, it is difficult to identify a plausible explanation for such an association; instead, an indirect association might be more likely, namely an association with a confounding variable. One possibility would be if degree of energy restriction during weight loss were a proxy measure for poor compliance (i.e. due to feeding extra food). Of course, such a mechanism is highly speculative, and further investigations would be needed, examining a greater number of variables.

A previous study in dogs had demonstrated that using a standard maintenance diet in the post-weight-loss period increases the risk of weight regain^(^^8^^)^. Unfortunately, it was not possible to examine this effect in the cats of the present study because there was a greater variability in the food fed, and many owners did not maintain an accurate record. Instead, therefore, we examined the effect of close monitoring, by comparing those owners who closely regulated their cat's intake (i.e. by accurately measuring food portions, not giving table scraps or treats, and maintaining records) with those who had not. Although simple logistic regression analysis suggested an effect, this factor did not remain in the final multiple regression model. Further work is recommended to determine the effect of post-weight-loss dietary management in a larger population of obese cats.

The findings in the present paper should be interpreted with caution due to a number of study limitations. First, although the obesity was naturally occurring in all cats, a referral population was studied, which might not be fully representative of the population of cats presenting to a first opinion practice. However, the advantage of using a referral population was the ability to conduct a more detailed case assessment, for example using dual-energy X-ray absorptiometry for body composition analysis, as well as more consistent weighing and monitoring during the programme, by a full-time veterinary nurse. A second limitation is potential bias, from the fact that this was a non-randomised cohort study. Thus, it is possible that an unmeasured confounding factor was responsible for the effects on regain.

A third concern was the fact that data on feeding practices after weight loss were limited. In a similar recent study, in dogs^(^^8^^)^, more detailed follow-up records were available. Also, since we relied on owners to provide information on feeding practices, accuracy of the data cannot be ensured. Future prospective studies should concentrate on recording more data from the weight maintenance phase to improve our understanding of the factors leading to weight regain.

A final limitation was the method used to determine the significance of any weight change after weight loss. We adopted a similar approach to that used in a recent dog study^(^^8^^)^, and defined limits in which deviation of body weight was acceptable after the weight loss period had been completed. This approach takes into account weigh scale inaccuracies and natural fluctuations in body weight that might result from differences in gut fill and hydration status^(^^8^^)^. The limit of ±5 % of target weight was applied, because this represents approximately half a unit on a nine-integer body condition score scale^(^^12^^,^^13^^)^, so that any difference is unlikely to be noted on a physical examination. Although it was assumed that weight fluctuations within this range should not be clinically relevant^(^^8^^)^, we cannot be absolutely certain of this. Similarly, we cannot be certain that regain >5 % of the target weight invariably has clinical consequences. Thus, further studies could be considered to determine acceptable limits for weight fluctuation in obese cats after weight loss, such that quality of life is maximised during both the short- and long terms.

### 

#### Conclusions

The present study has demonstrated that almost half of obese client-owned cats will regain weight following a successful weight loss programme, with a large proportion regaining more than half of the original weight loss. Weight regain is most likely in younger cats. These findings highlight the importance of continuing to monitor cats after weight loss is completed to ensure that the health benefits of weight loss are maintained.
